# Preoperative sonographic diagnosis of McSwain type V appendiceal intussusception: A case report (with video)

**DOI:** 10.1097/MD.0000000000023452

**Published:** 2020-12-04

**Authors:** Xing-xing Duan, Ya Peng, Liu Yang, Wen-juan Chen, Xiang-lian Tang

**Affiliations:** a Department of Ultrasound; b Department of paediatric surgery, Hunan Children's Hospital, Changsha, Hunan Province, China.

**Keywords:** appendix, child, diagnosis, intussusception, ultrasound

## Abstract

**Rationale:**

Appendiceal intussusception is a rare disease. The definite preoperative diagnosis of appendiceal intussusception is rare and challenging. Here, we present a case of McSwain type V appendiceal intussusception in a 10-year-old boy. To our best knowledge, this is the first case report of a type V appendiceal intussusception that was preoperatively confirmed with sonography. Here, we have described in detail the ultrasound features and differential diagnosis of this rare disease.

**Patient concerns:**

A 10-year-old boy presented with 3 days of recurrent intermittent mild abdominal pain. The result of ultrasonography suggested an ileocolic intussusception and a therapeutic air-contrast enema was requested to reduce the intussusception but failed at a local hospital.

**Diagnoses:**

Physical exam revealed mild tenderness in the lower right quadrant of the abdomen. However, ultrasonography showed a target-sign in cross section and a finger-like appearance in the longitudinal view. A diagnosis of McSwain type V appendiceal intussusception was made.

**Interventions:**

The patient underwent an appendectomy after successful manual reduction on laparotomy. The appendix was successfully resected.

**Outcomes:**

Intraoperatively, the appendix was completely inverted in the cecum, and the preoperative sonographic findings were confirmed. During follow-up, there were no signs of recurrence.

**Lessons:**

Pre-operatively, on ultrasound a type V appendiceal intussusception is usually misdiagnosed as an ileocolic intussusception. Radiologists must execute caution to avoid over reliance on the sonographic findings of intussusception, especially when there is a mismatch with clinical symptoms. It is especially important to accurately understand the surgical-anatomic configuration of type V appendiceal intussusception that creates a “target-sign” and a “finger-like” layout on ultrasonography.

## 1 Introduction

Appendiceal intussusception is a rare disease characterized by invagination of the appendix into the cecum to various degrees. The clinical presentation of appendiceal intussusception is extremely variable and non-specific. A definite preoperative diagnosis of appendiceal intussusception is more rare and challenging.^[1]^ Most of the literature regarding appendiceal intussusception discusses the surgical and colonoscopic approaches for treatment of this condition.^[2,3]^ The existing radiology literature on appendiceal intussusception largely discusses about computed tomography,^[4]^ barium enema and double-contrast enema^[5]^ findings. There is very limited literature describing the appearance of appendiceal intussusception on sonography.^[6]^ Further, it is not detailed enough in characterizations and differential diagnosis of appendiceal intussusception. To our best knowledge, this is the first case report of type V appendiceal intussusception that was based on preoperative diagnosis using only sonography. Here, we describe in detail the ultrasound features and the differential diagnosis of this rare disease. We also review the literature on appendiceal intussusception. We hope that our report will facilitate the preoperative diagnosis of appendiceal intussusception.

## 2 Case report

A 10-year-old boy presented with 3 days of recurrent intermittent mild abdominal pain, but no fever, vomiting, abdominal distention, obstipation, or bloody stool. One day prior to admission, abdominal ultrasonographic examination was performed at a local hospital and the result was suggestive of an ileocolic intussusception. A therapeutic air-contrast enema was requested at the local hospital to reduce the intussusception but failed. The patient was presented to our hospital for further evaluation and treatment.

**Figure 1:**
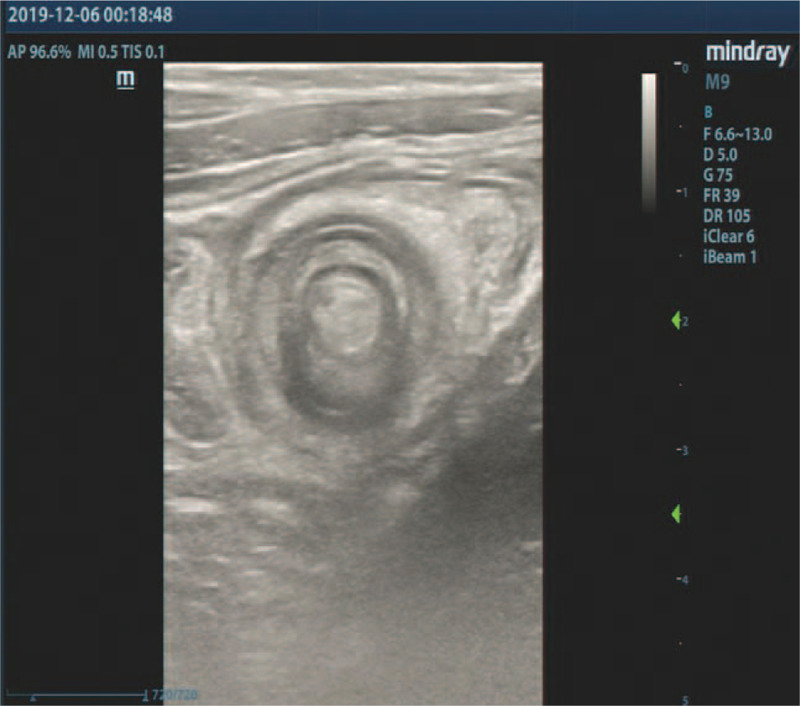
Axial sonogram of the intussusception shows a multiconcentric ring sign with a multilayer appearance.

**Figure 2:**
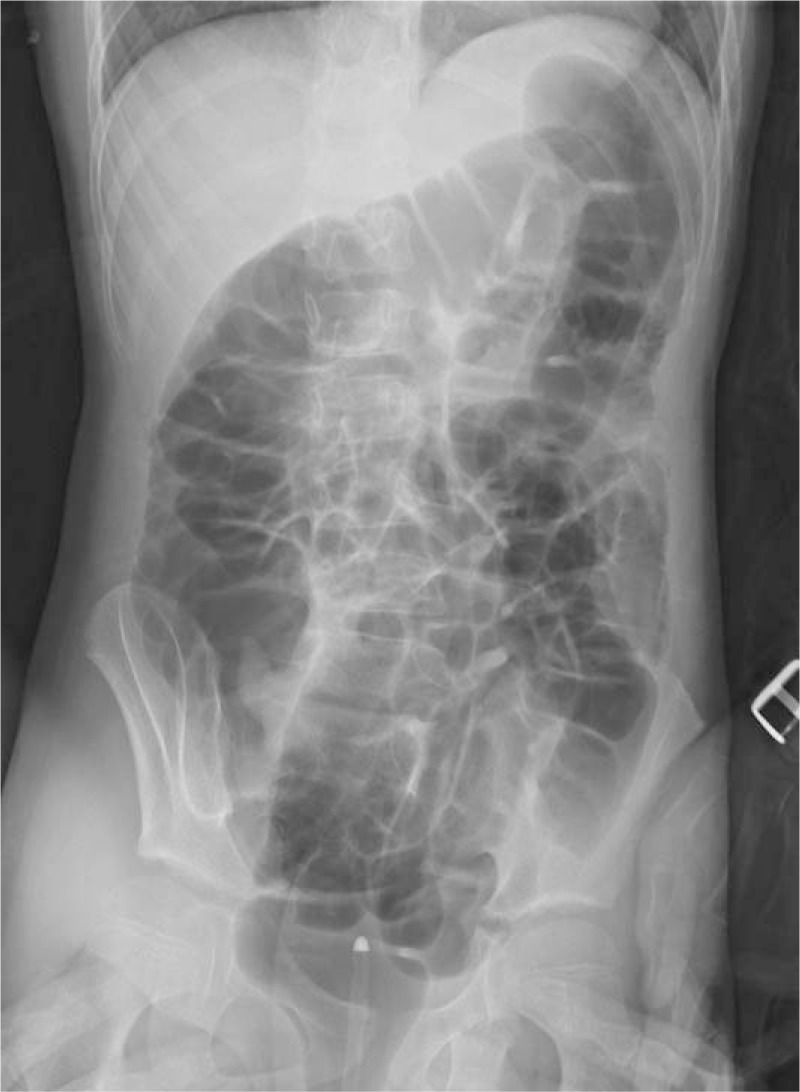
Large amount of air reflux seen in the terminal ileum and small bowel under the fluoroscopy, reveals successful reduction.

On the day of admission, physical examination revealed mild tenderness on deep palpation but no sign of peritoneal irritation. A mass was palpable in the lower right quadrant of the abdomen and there were reduced bowel sounds on auscultation (once per minute). The results of routine blood tests, including white blood cell count, C-reactive protein and hemoglobin concentration level were within normal limits. A plain abdominal film was within normal limits. To decide whether the patient had an acute abdomen (intussusceptions or appendicitis) or not, abdominal ultrasound was performed using a Mindray M9 scanner, a 6.6 to 3 MHz L12-4 s linear–array transducer (Mindray Medical Ultrasound Systems, Shenzhen, China). The sonographic findings were suggestive of intussusception (Fig. 1), and an air-contrast enema was performed to reduce intussusception. Fluoroscopy demonstrated an air reflux into the terminal ileum and small bowel, which is considered to be a sign of successful reduction (Fig. 2). The patient was placed under close observation in the surgical ward after the reduction. However, the patient continued to complain of abdominal pain and discomfort during the observation period of 8 hours. Repeated abdominal ultrasonography demonstrated ongoing intussusception. The sonographic appearance (including size and structure) of intussusception was similar to the previous scan findings. Repeated air-contrast enema reduction was performed which was successful. However, 4 hours after the second reduction at our institution, the patient still showed no signs of improvement. Considering the discrepancy seen among the clinical presentation, abdominal sonography (that was done twice) and air-contrast enema findings, the pediatric surgeon requested to repeat the abdominal ultrasound examination.

**Figure 3:**
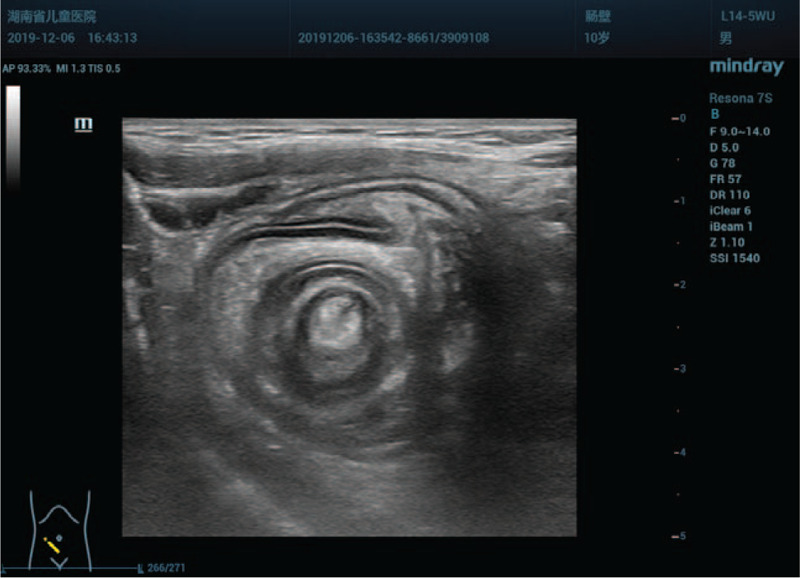
Axial sonogram of the right lower abdominal quadrant shows a target sign, the inner ring with a prominent central hyperecho representing the inverted appendix and mesoappendix.

**Figure 4:**
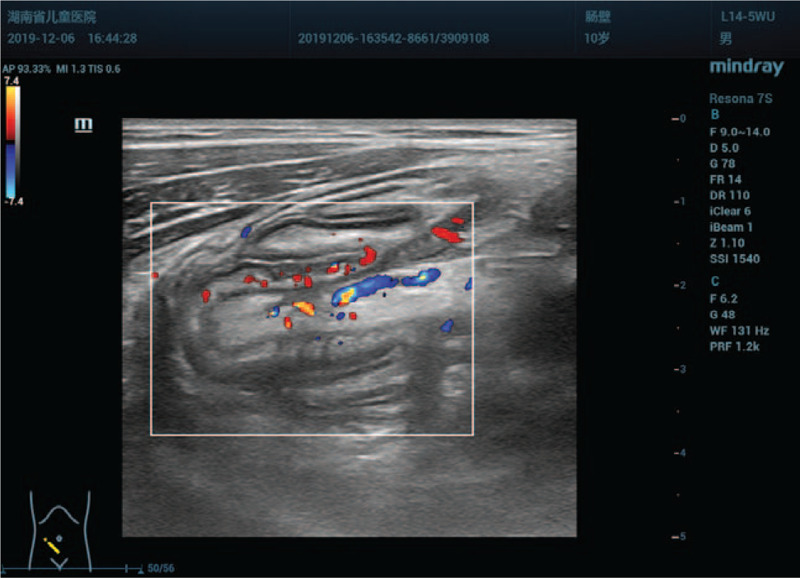
Longitudinal view of the intussusceptum shows a finger-like appearance, and color Doppler flow imaging shows an artery supplied to the intussusceptum.

**Figure 5:**
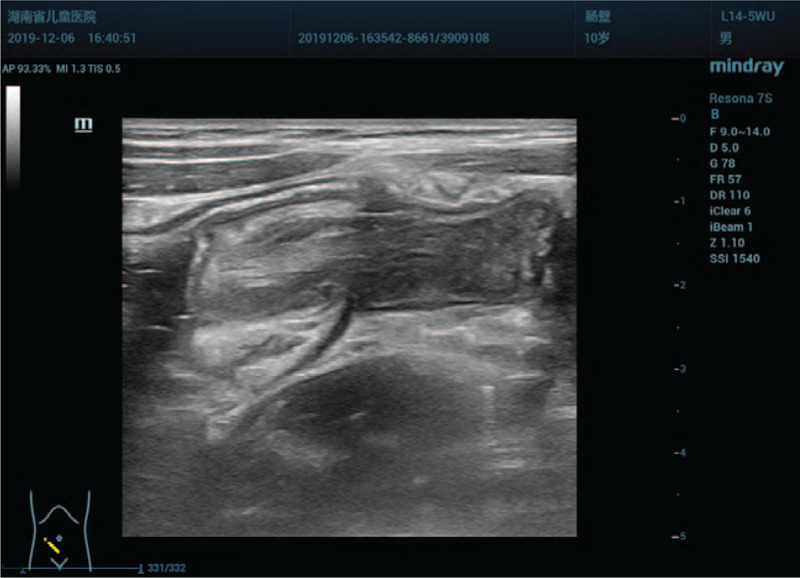
Longitudinal view of the normal ileocecal valve and terminal ileum were observed in the right lower quadrant of the abdomen.

This time, a careful examination was performed with Resona 7S scanner, a 9-14 MHz L14-5WU linear–array transducer (Mindray Medical Ultrasound Systems, Shenzhen, China). The results showed that there was a permanent target-sign suggestive of intussusception on transverse image in the right lower quadrant of the abdomen which was similar to previous finding. However, the sonographic appearances were unusual for a classic ileocolic intussusception. Firstly, in cross section, the diameter of the round intussusceptum was 15 mm, and the center was homogeneous, hyperechoic with absence of intralesional lymph nodes. There was no abutting wall between the intussusceptum and intussuscipiens (Fig. 3). In the longitudinal view, the intussusceptum had a finger-like appearance which was blind-ended and protruding into the cecum. The base of the intussusceptum continued into the tip of the cecum, and the central homogeneous hyperechoic region extending from the interior of the intussusceptum to the medial of the cecum. A blood vessel was observed in the homogeneous hyperechoic region. Color Doppler flow imaging confirmed evidence of arterial flow to the intussusceptum (Fig. 4). Secondly, the appendix could not be identified around the cecum of this patient, who had no history of appendectomy. Thirdly, a normal ileocecal valve and terminal ileum were observed in the right lower quadrant of the abdomen (Fig. 5), which excluded an ileocolic intussusception (see Video, Supplemental Video which demonstrates the appendix was completely inverted in the cecum). We further carefully analyzed the sonographic appearances. The finger-like structure was interpreted as an appendix which was completely invaginated into the cecum. The mesoappendix was thought to be responsible for the central homogeneous hyperecho. The artery was identified as arteriae appendicularis, and the ileocecal valve was normal. Therefore, the clinical presentation, the abdominal sonographic appearances and the air-contrast enema reduction findings could be correctly interpreted. A positive preoperative diagnosis of McSwain type V appendiceal intussusception was established using sonography.

**Figure 6:**
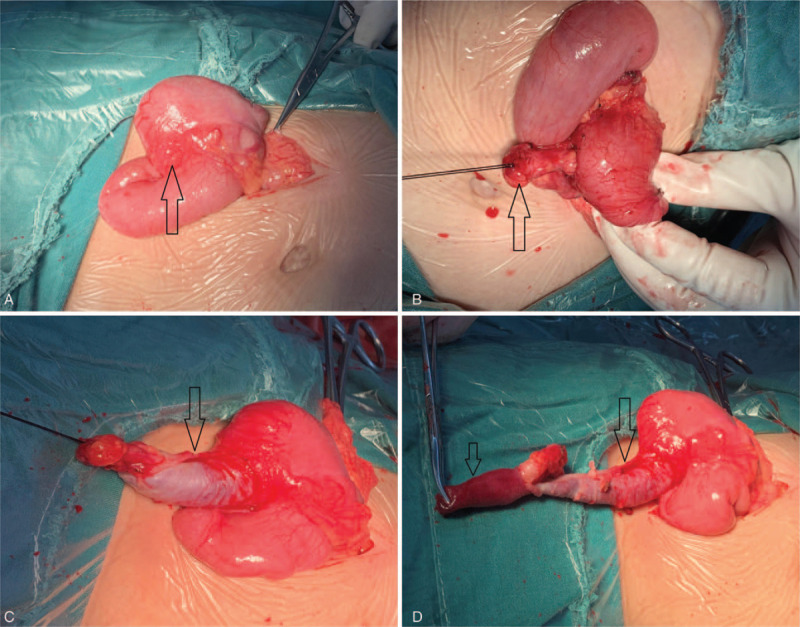
Intraoperative photographs. A; shows complete inversion of the appendix into the cecum (arrow). B; shows the mesoappendix was dragged out from the cecum (arrow). C; shows the proximal appendix was dragged out (arrow). D; shows the appendix was dragged out completely. The appendix was torn during manual reduction. The proximal appendix wall was significantly thickened (long arrow) and the distal appendiceal mucosa has a dark-red appearance (short arrow).

Two days following admission, the patient underwent surgery. Taking into consideration that the McSwain type V appendiceal intussusception was not amenable for laparoscopic reduction, and to rule out other appendicular intramural or intraluminal lesions, especially malignant lesion and congenital intestinal malformation, a laparotomy was selected. The patient was given general anesthesia and a 5 cm long vertical incision was made beside the right rectus abdominis. Intraoperatively, the appendix was found to be completely inverted in the cecum and the preoperative diagnosis was thus confirmed. An appendectomy was performed after successful manual reduction (Fig. 6). There was no evidence of an appendiceal mass. At gross pathology, the resected appendix measured 9 cm in length and 1.5 cm in width, the proximal appendix wall was significantly edematous, thickened and indurated. The distal appendiceal mucosa had a dark-red appearance and hemorrhage was identified. Histologic sections showed a hemorrhagic necrosis and inflammatory cell infiltrate within the distal appendix. Postoperative recovery was uneventful. The patient was discharged home on the 5^th^ day postoperatively and has been symptom-free ever since.

## 3 Discussion

Appendiceal intussusception is a rare disease. As per Collina's study, which analyzed 71,000 appendices obtained from surgical patients and autopsies estimated that the incidences of appendiceal intussusception was 0.01%.^[7]^ Earlier literature suggested that the incidences of appendiceal intussusception are more common in the children^[8]^; however, a recent review reported that the adults are more commonly affected (76% versus 24%).^[9]^ The clinical presentation of appendiceal intussusception include intermittent abdominal pain, fever, vomiting, and bleeding per-rectum, however, it is often vague and it varies from being asymptomatic to presenting with chronic abdominal pain.^[10]^ In some patients, the symptoms may mimic acute appendicitis.^[11]^ In the present case, the patient was hospitalized because of recurrent intermittent abdominal pain without other symptoms.

Appendiceal intussusception has numerous underlying causes. The most common causes of appendiceal intussusception in children are appendiceal inflammation.^[12]^ Other causes include foreign body, lymphoid hyperplasia, parasites, polyps, endometriosis, and mucinous cystadenoma.^[11,13]^ In this case, inflammatory changes without organic disease were not only observed in gross pathology, but also in microscopic pathology.

Appendiceal intussusception is classified based on the region of the appendix involved in intussusception by McSwain.^[14]^ Type I: The tip of the appendix (intussusceptum) is invaginated into the body of the appendix (intussuscipiens). Type II: The invagination begins at some point along the length of the appendix. The adjacent tissue forms the intussuscipiens. Type III: The intussusception starts at the root of the appendix and the cecum is the intussuscipiens. Type IV: The proximal appendix (intussusceptum) is invaginated into the distal appendix (intussuscipiens), this is a retrograde intussusception. Type V: The completely inverted appendix (intussusceptum) is invaginated into the cecum (intussuscipiens). Our case was classified as type V appendiceal intussusception and only inflammatory changes without organic disease were observed in pathology.

The treatments of appendiceal intussusception, including the selection of surgical and operative methods, differ significantly depending on its type and whether the causative disease is malignant or benign.^[15]^ Therefore, the exact preoperative diagnosis is vital. Radiologic studies may also be helpful in diagnosis. According to the literature, the appearance of appendiceal intussusception on barium enema has been described as a “coiled-spring” or “spiral shell” appearance, with lack of filling in the appendix.^[16]^ Air-contrast enema describes appendiceal intussusception as a “finger-like projection” outlined by air within the cecum.^[5]^ The CT scan may be helpful in diagnosis of the appendiceal intussusception as it reveals excellent anatomic details and reconstructions. A target, layered and sausage-shaped appearance may be observed on CT.^[17]^ A mushroom or polypoid caecal lesion may be observed on colonoscopy.^[18]^ There is some literature regarding the appearance of appendiceal intussusception on sonography,^[6]^ but it is not detailed enough in characterizations and differential diagnosis.

In the present case, we found that the target-sign with homogeneous hyperechoic center on transverse images and the finger-like appearance with homogeneous hyperechoic interior on longitudinal images were the ultrasound features of type V appendiceal intussusception. The patient was accurately diagnosed with type V appendiceal intussusception using preoperative sonography. An appendectomy after reduction of the appendix on laparotomy was selected as the surgical strategy. Furthermore, in this case, we selected the abdominal ultrasound as the tool for diagnosing the appendiceal intussusception on the basis of following advantages. Firstly, ultrasonography is a simple, convenient, repeatable and the most commonly used examination method for intussusception investigation with high sensitivity, and specificity.^[19]^ Secondly, there is no risk of exposure to ionizing radiation and allergy to contrast material compared with computed tomography. Moreover, it is a noninvasive method and needs no special preparation compared with colonoscopy. Additionally, colonoscopy also has the possibility of misdiagnosis and the risk of mistreatment.^[20]^ Even if there is a possibility of misdiagnosis, we still recommend to use ultrasound in such cases in children.

Notably, the target-sign appearance of intussusception mislead 3 radiologists (including the ones working at other institution) resulting in inappropriate treatment strategy. Therefore, the differential diagnosis is important. The sonographic appearances of appendiceal intussusception differed from ileocolic intussusception. In classic ileocolic intussusception, the target-sign shows multiple concentric hypoechoic and hyperechoic rings. The outermost ring is formed by colon (intussuscipiens). While, the innermost ring is constituted by the ileocecal valve and terminal ileum (intussusceptum). The middle layer consists of abutting intestinal wall. Between the middle layer and innermost ring, there are some hypoechoic enlarged mesenteric lymph nodes and hyperechoic mesentery. Occasionally, the cross-sectional image of appendix is observed in the side of the innermost ring.^[21,22]^ Whereas, in the type V appendiceal intussusception, according to our case, the innermost ring constituted of inverted appendix, no enlarged mesenteric lymph nodes were found in the target-sign mass, and the ileocecal valve was normal. An inverted Meckel diverticulum with or without intussusception may also have a finger-like appearance. However, it is located at distal ileum, not cecum,^[6,23]^ and the appendix is normal. The sonographic features of other diseases of the appendix should not pose a problem in differential diagnosis for no other diseases mimic the classic target-sign in sonography.

## 4 Conclusions

We present here a case of McSwain type V appendiceal intussusception in a 10-year-old boy who was diagnosed using ultrasonography and explain the sonographic image on the basis of the surgical-anatomic basis. Radiologists should execute caution while diagnosing intussusception using sonography, especially when the sonography has a mismatch with clinical symptoms. It is especially important to have accurate understanding of the surgical-anatomic configuration of the type V appendiceal intussusception creating the “target-sign” and “finger-like” layout on ultrasonography.

## Acknowledgments

We are thankful to the editors for the language help and the reviewer's comments and advice.

## Author contributions

**Conceptualization:** Xing-xing Duan.

**Data curation:** Xing-xing Duan, Ya Peng, Liu Yang, Xiang-lian Tang.

**Formal analysis:** Xing-xing Duan.

**Investigation:** Xing-xing Duan.

**Methodology:** Xing-xing Duan.

**Project administration:** Xing-xing Duan.

**Resources:** Wenjuan Chen.

**Writing – original draft:** Xing-xing Duan, Ya Peng, Liu Yang, Wenjuan Chen, Xiang-lian Tang.

**Writing – review & editing:** Xing-xing Duan, Ya Peng, Liu Yang, Wenjuan Chen, Xiang-lian Tang.

## Supplementary Material

Supplemental Digital Content
